# Supplementary material for: Structures of human prostaglandin F2α receptor reveal the mechanism of ligand and G protein selectivity
Source: Nat Commun. 2023 Dec 8;14:8136. doi: 10.1038/s41467-023-43922-8 (PMC10709307; doi:10.1038/s41467-023-43922-8)
Supplement: Supplementary file 1 — Supplementary Information [file 41467_2023_43922_MOESM1_ESM.pdf]

Structures of human prostaglandin $F_{2\alpha}$ receptor reveal the mechanism of ligand and G protein selectivity

Xiuqing Lv[1,8], Kaixuan Gao[2,3,8], Jia Nie[1,8], Xin Zhang[2,3], Shuhao Zhang[2,3], Yinhang Ren[2,3], Xiaoou Sun[3,4], Qi Li[5], Jingrui Huang[1], Lijuan Liu[1], Xiaowen Zhang[1], Weishe Zhang[1,6#], Xiangyu Liu[2,3,7#].

Affiliations:

[1] Department of Obstetrics, Xiangya Hospital Central South University, Changsha, China.

[2] State Key Laboratory of Membrane Biology, Tsinghua-Peking Center for Life Sciences, School of Pharmaceutical Sciences, Tsinghua University, Beijing, China.

[3] Beijing Frontier Research Center for Biological Structure, Beijing Advanced Innovation Center for Structural Biology, Tsinghua University, Beijing, China.

[4] School of Medicine, Tsinghua University, Beijing, China.

[5] Reproductive Medicine Center, Xiangya Hospital Central South University, Changsha, China.

[6] Hunan Engineering Research Center of Early Life Development and Disease Prevention, Changsha, China.

[7] Beijing Key Laboratory of Cardiovascular Receptors Research, Peking University, Beijing, China

[8] These authors contributed equally: Xiuqing Lv, Kaixuan Gao, Jia Nie

[#] Co-corresponding authors

Weishe Zhang, email: zhangweishe@yeah.net

Xiangyu Liu: liu_xy@mail.tsinghua.edu.cn

Supplementary figure

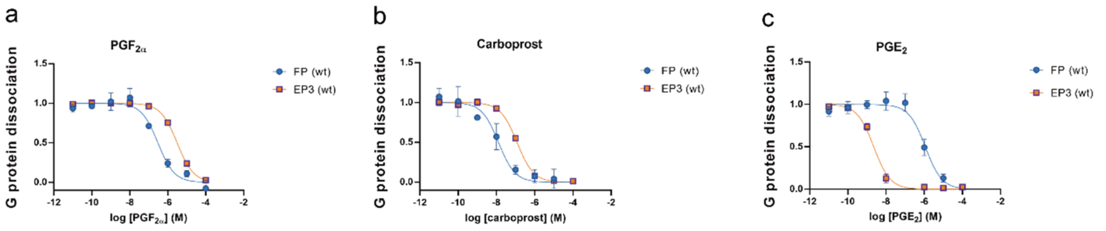

	pEC50
FP (wt)	6.52±0.05
EP3 (wt)	5.51±0.01

	pEC50
FP (wt)	7.89±0.04
EP3 (wt)	6.92±0.03

	pEC50
FP (wt)	5.94±0.04
EP3 (wt)	8.65±0.03

Supplementary Fig. 1 The selectivity of $PGF_{2\alpha}$, carboprost, and PGE_2 toward FP receptor and EP3 receptor. a-c The $PGF_{2\alpha}$ (a), caroprost (b) and PGE_2 (c) activation profiles of FP receptor and EP3 receptor revealed by NanoBiT assay. Data are represented as mean ± SD of three independent experiments. Source data are provided as a Source Data file.

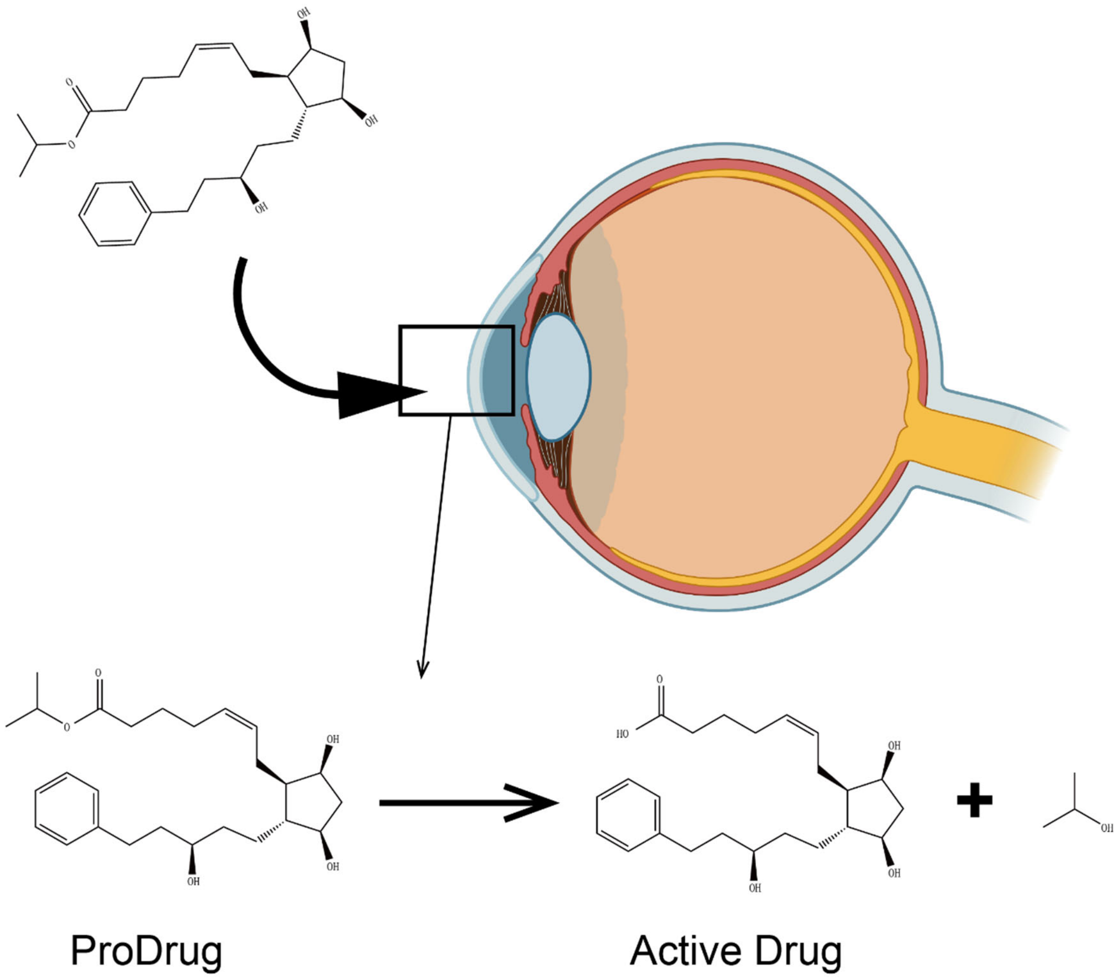

Supplementary Fig. 2 Prodrug to drug activation of latanoprost in eyes. The figure is created by BioRender**.**

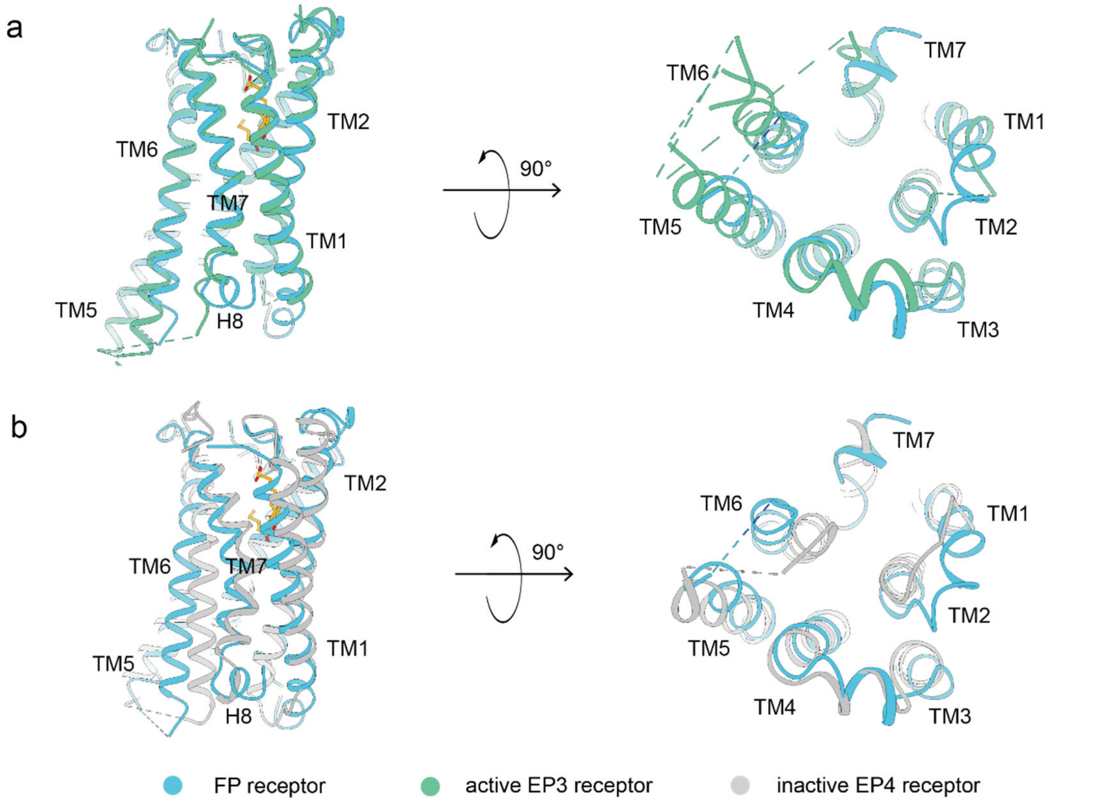

Supplementary Fig. 3 The active-state conformation of FP receptor compared to active-state EP3 receptor and inactive-state EP4 receptor structures. a Structure alignment of FP-miniG$_{s/q70iN}$ structure with active EP3 structure (PDB: 6AK3). **b** Alignment of FP-miniG$_{s/q70iN}$ structure with inactive EP4 structure (PDB: 5YWY). FP receptor, EP3 receptor and EP4 receptor are colored blue, grey blue and green respectively.

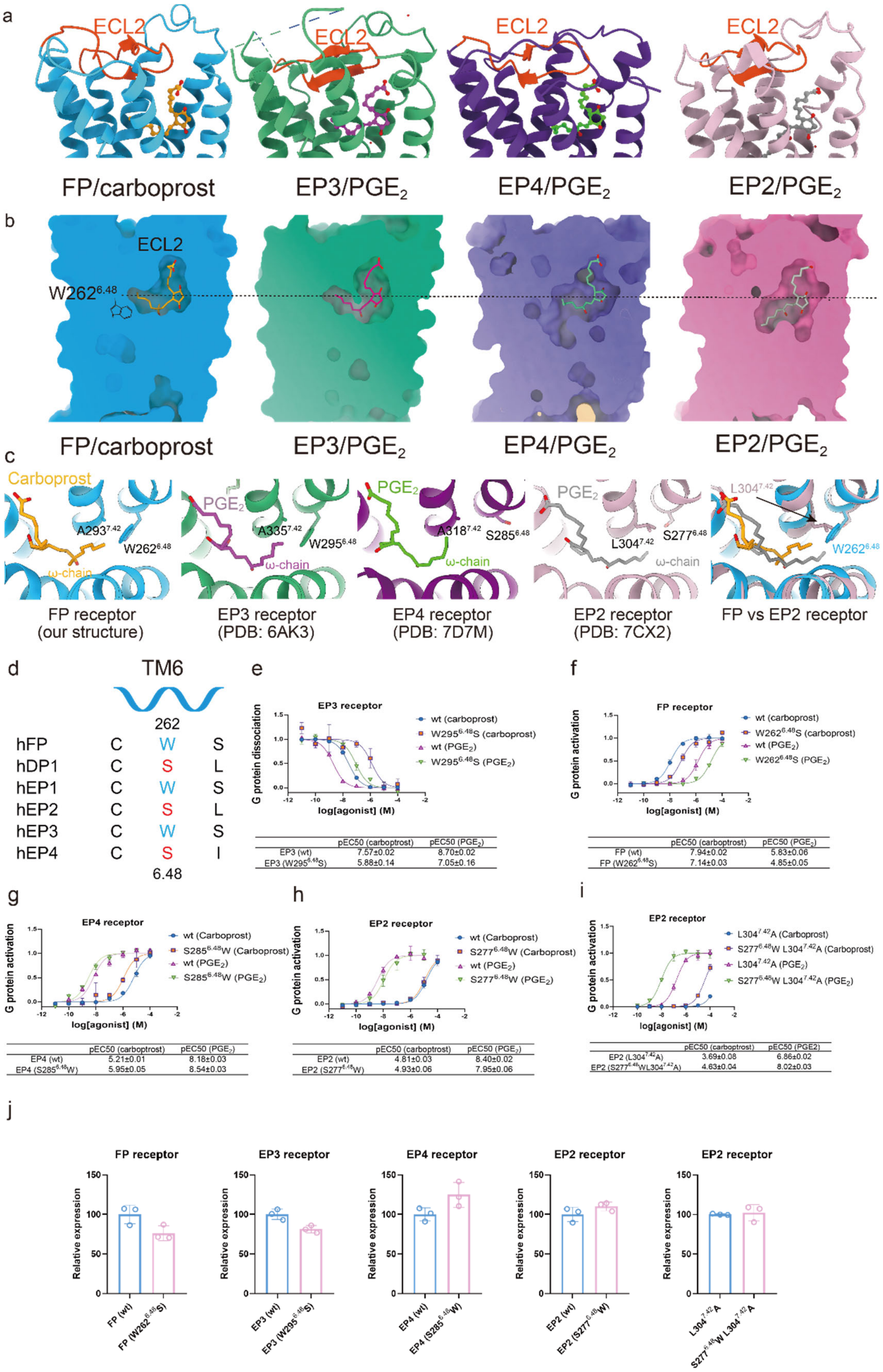

Supplementary Fig. 4 Comparison of the orthosteric pockets between FP receptor, EP3 receptor, EP2 receptor and EP4 receptor. a Hairpin structure formed

by ECL2 (red) in prostaglandin receptors. **b** FP receptor and EP3 receptor have more compact orthosteric pockets compared to the other two structures due to the $W^{6.48}$ and $S^{6.48}$ difference. **c** The comparison of residues around ω-chain in FP, EP3, EP4 and EP2 receptor. **d** Sequence alignment suggests the differences on the toggle switch $W/S^{6.48}$. **e** Carboprost and PGE_2-mediated Gi signaling on wild-type EP3 receptor and EP3_W295$^{6.48}$S using a NanoBiT assay. **f-i** Carboprost and PGE_2-mediated signaling on wild-type FP receptor and FP_W262$^{6.48}$S (f), EP4 receptor and EP4_S285$^{6.48}$W (g), EP2 receptor and EP2_S277$^{6.48}$W (h) as well as EP2_L304$^{7.42}$A and EP2_ S277$^{6.48}$W/L304$^{7.42}$A double mutation (i) using a GloSensor cAMP assay. **j** The expression levels of FP, EP2, EP3, and EP4 receptor and their mutants in cos7 measured by cell surface staining. Data are represented as mean ± SD of three independent experiments. Source data are provided as a Source Data file.

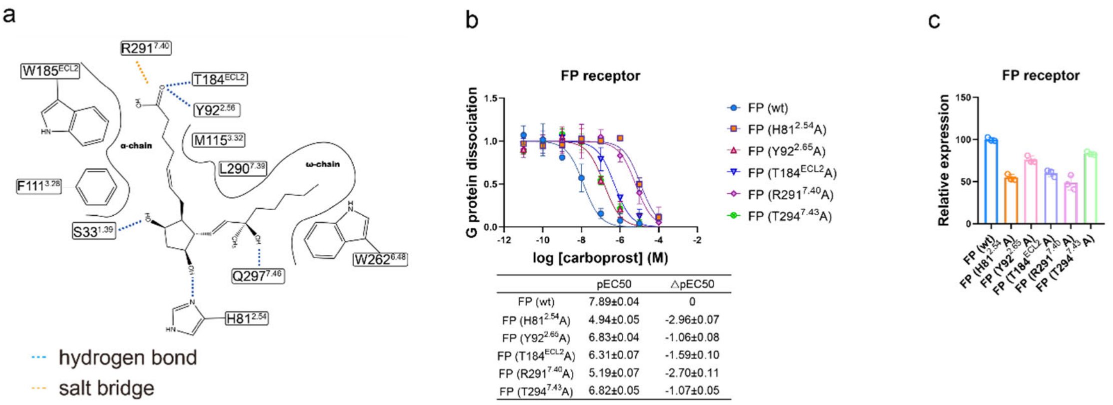

Supplementary Fig. 5 The carboprost binding pocket. a Two-dimensional schematic depiction of the carboprost binding pocket on the FP receptor. H-bonds and salt bridges are displayed as blue and orange dash lines, respectively. Solid lines represent hydrophobic interfaces. **b** The carboprost activation profiles of FP receptor and the binding pocket mutants revealed by NanoBiT assay. **c** Expression levels of FP receptor and its mutants measured by cell surface staining. Data are given as mean ± SD from 3 independent experiments. Source data are provided as a Source Data file.

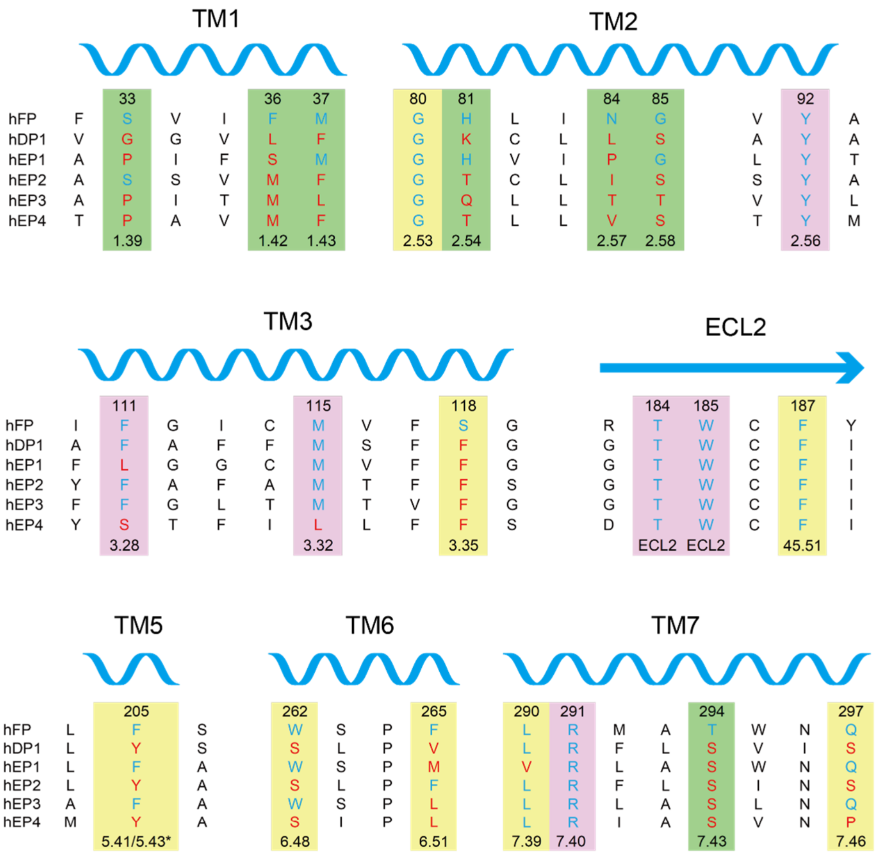

Supplementary Fig. 6 Sequence alignment of orthosteric pocket residues in prostaglandin receptors. The residues that directly interact with carboprost in the FP receptor (defined by within 4 Å distance) are colored blue. The equivalent residues in other prostaglandin receptors are also colored blue if they are identical to those in FP receptor, otherwise, the residues are colored red. Green box indicates the residues interact with the F ring of the ligand, pink box indicates residues interact with α-chain and yellow box indicates residues interact with the ω-chain. *Structure alignment suggest residues 5.41 of FP receptor, DP1 receptor, EP1 receptor and EP2 receptor are in the same position as residues 5.43 of EP3 receptor and EP4 receptor (Ballesteros-Weinstein numbering based on GPCRDB). The structures include: FP receptor structure (this study), DP1 receptor (AlphaFoldDB: Q13258), EP1 receptor structure (AlphaFoldDB: P34995), EP2 receptor structure (PDB: 7CX2), EP3 receptor (PDB: 7WU9) and EP4 receptor (PDB: 7D7M).

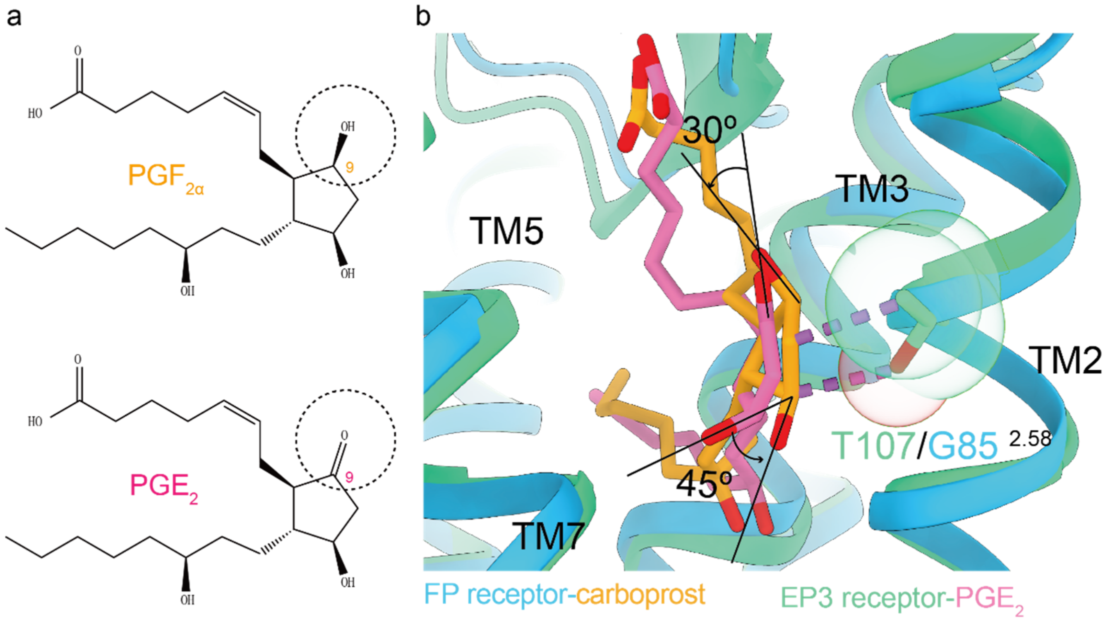

Supplementary Fig. 7 The effect of 9-hydroxyl group or 9-carbonyl group on ligand binding to the prostaglandin receptors. a The chemical structures of $PGF_{2α}$ and PGE_2. **b** Comparison of ligand binding mode between FP-carboprost and EP3-PGE_2. Arrows show the difference in 9-group and 11-group orientation. The color code for the receptors is as follows: FP receptor (blue), EP3 receptor (green), carboprost (orange), PGE_2 (pink).

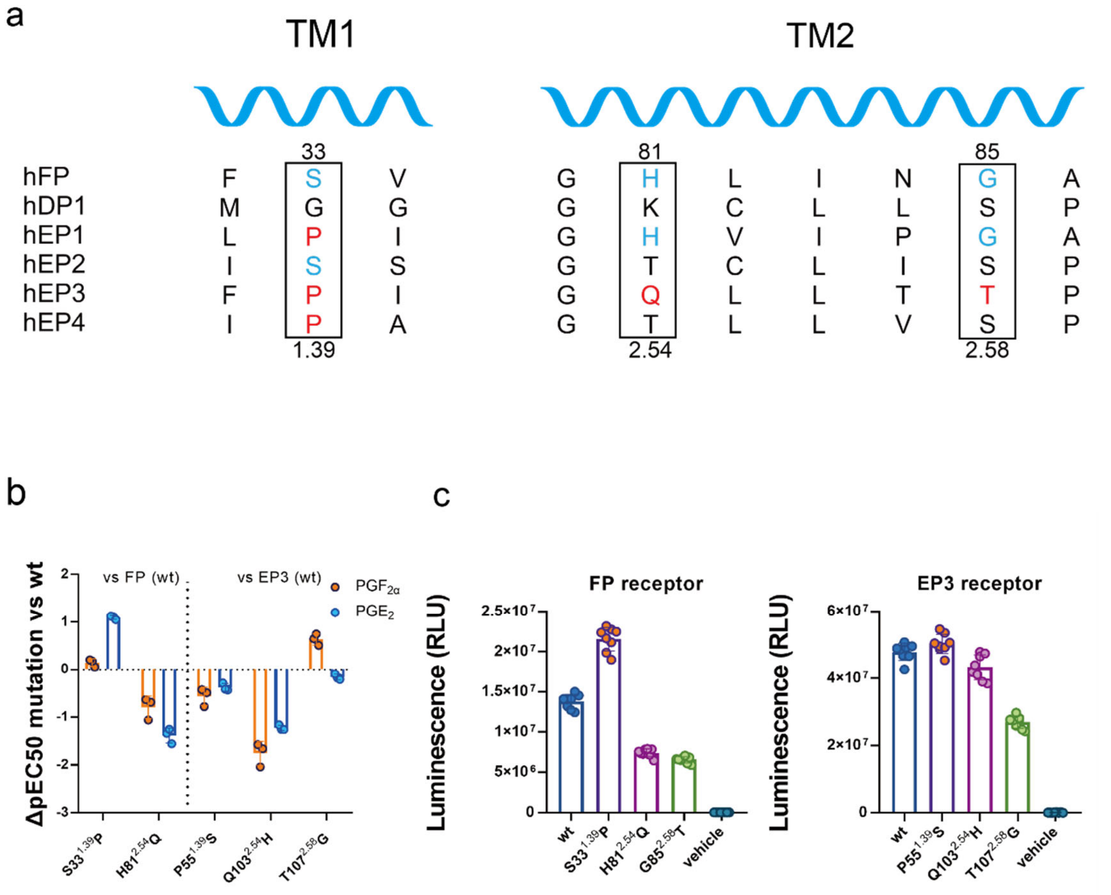

Supplementary Fig. 8 Mutagenesis studies of key residues involving in cyclopentane ring interaction in the FP receptor or EP3 receptor. **a** Sequence alignment of residues involved in cyclopentane ring interaction in the prostaglandin receptor family. The residues that involved the formation of hydrogen bonds in the FP receptor are colored blue while the equivalent residues in EP3 receptor residues are colored red. The equivalent residues in other receptors are colored in blue if they are identical to those in FP receptor and red if they are identical with those in EP3 receptor, otherwise they are in black. **b** The FP (S33$^{1.39}$P), FP (H81$^{2.54}$Q), EP3 (P55$^{1.39}$S), EP3 (Q103$^{2.54}$H) and EP3 (T107$^{2.58}$G) have different effects on the potency of $PGF_{2\alpha}$ and PGE_2 as shown by ΔpEC50 between the mutant and the wt receptors. **c** Expression levels of FP receptor, EP3 receptor, and their mutants measured by HiBiT assay. Data are given as mean ± SD from 3 (b) or 8 (c) independent experiments. Source data are provided as a Source Data file.

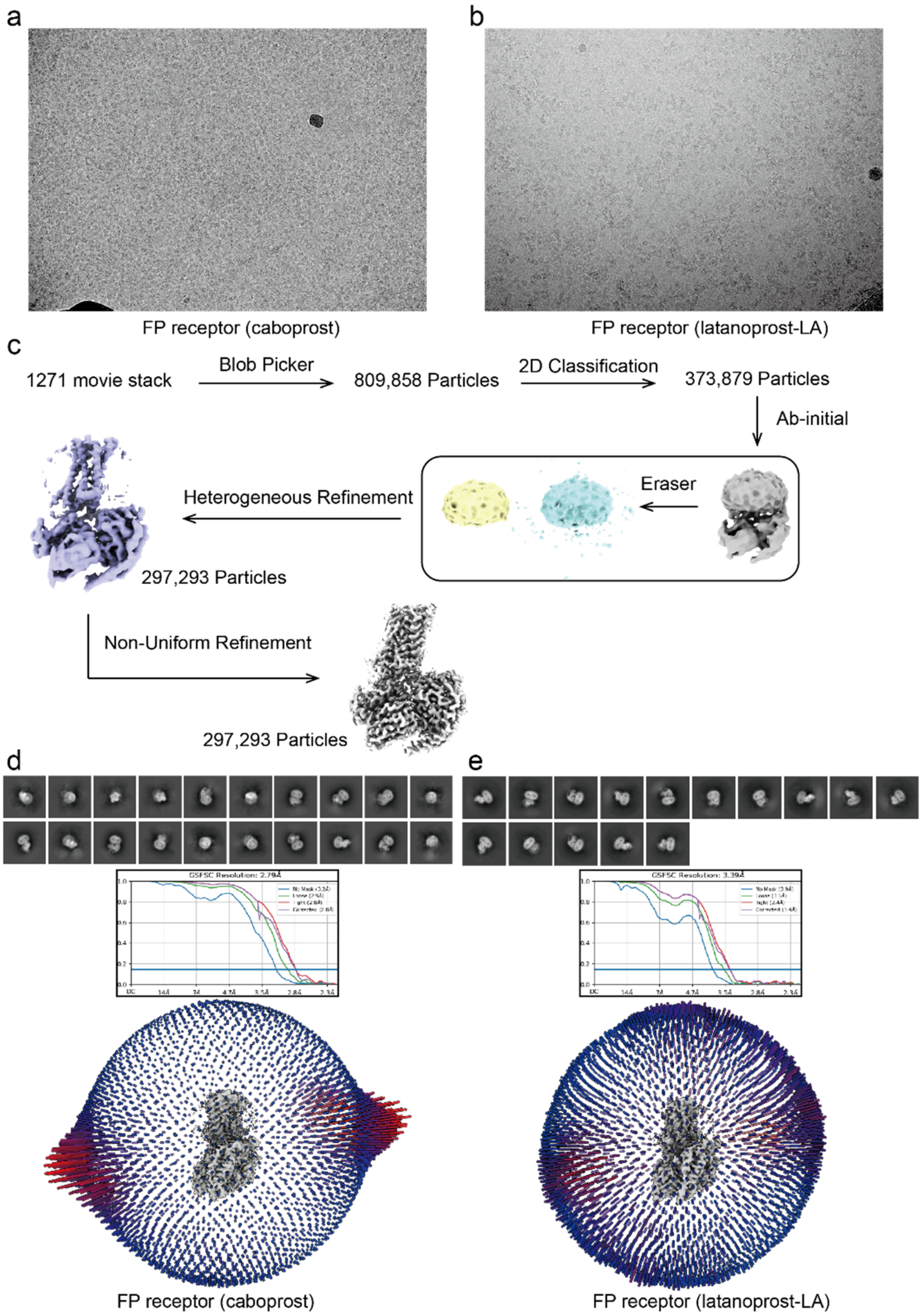

Supplementary Fig. 9 The cryo-EM data processing of FP-miniG$_{s/q70iN}$-G$\beta_1\gamma_2$-Nb35 complex. **a** A representative micrograph of FP-miniG$_{s/q70iN}$-G$\beta_1\gamma_2$-Nb35 complex binding with carboprost tromethamine. **b** A representative micrograph of FP-miniG$_{s/q70iN}$-G$\beta_1\gamma_2$-Nb35 complex binding with latanoprost-FA. **c** Cryo-EM data processing workflow for FP-miniG$_{s/q70iN}$-G$\beta_1\gamma_2$-Nb35 complex. **d** 2D classification and the "Gold-standard" FSC curve for FP-miniG$_{s/q70iN}$-G$\beta_1\gamma_2$-Nb35 binding with carboprost tromethamine. **e** 2D classification the "Gold-standard" FSC curve for FP-miniG$_{s/q70iN}$-G$\beta_1\gamma_2$-Nb35 binding with latanoprost-FA.

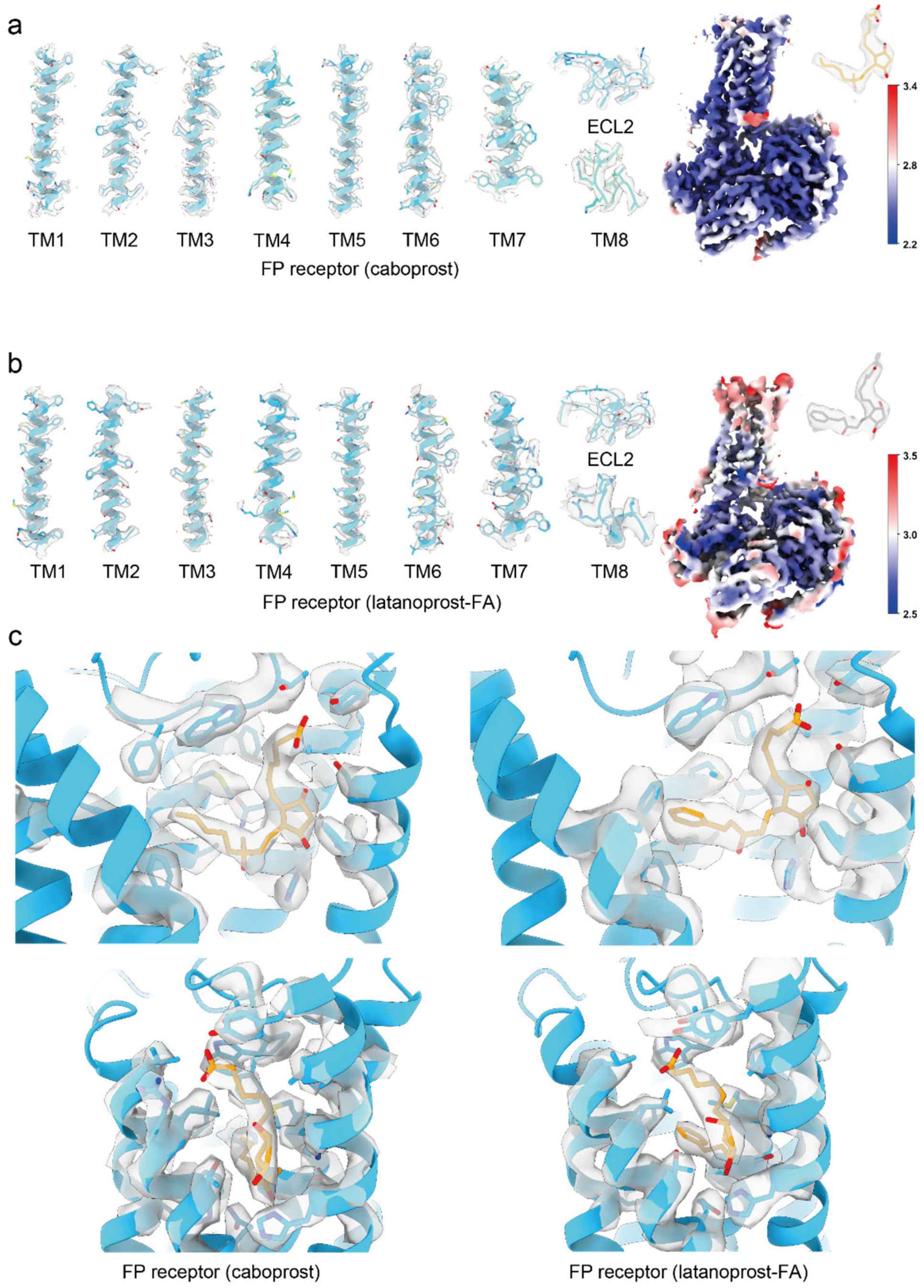

Supplementary Fig. 10 The cryo-EM densities of FP-miniG$_{s/q70iN}$-Gβ$_1$γ$_2$-Nb35 complex. a, b Unsharpened cryo-EM density map for individual FP receptor helices, ECL2 and H8, as well as the local resolution of FP-miniG$_{s/q70iN}$-Gβ$_1$γ$_2$-Nb35 bound with carboprost tromethamine or latanoprost-FA. **c** Electron densities of the binding pocket residues and the ligands shown at the same sigma level.

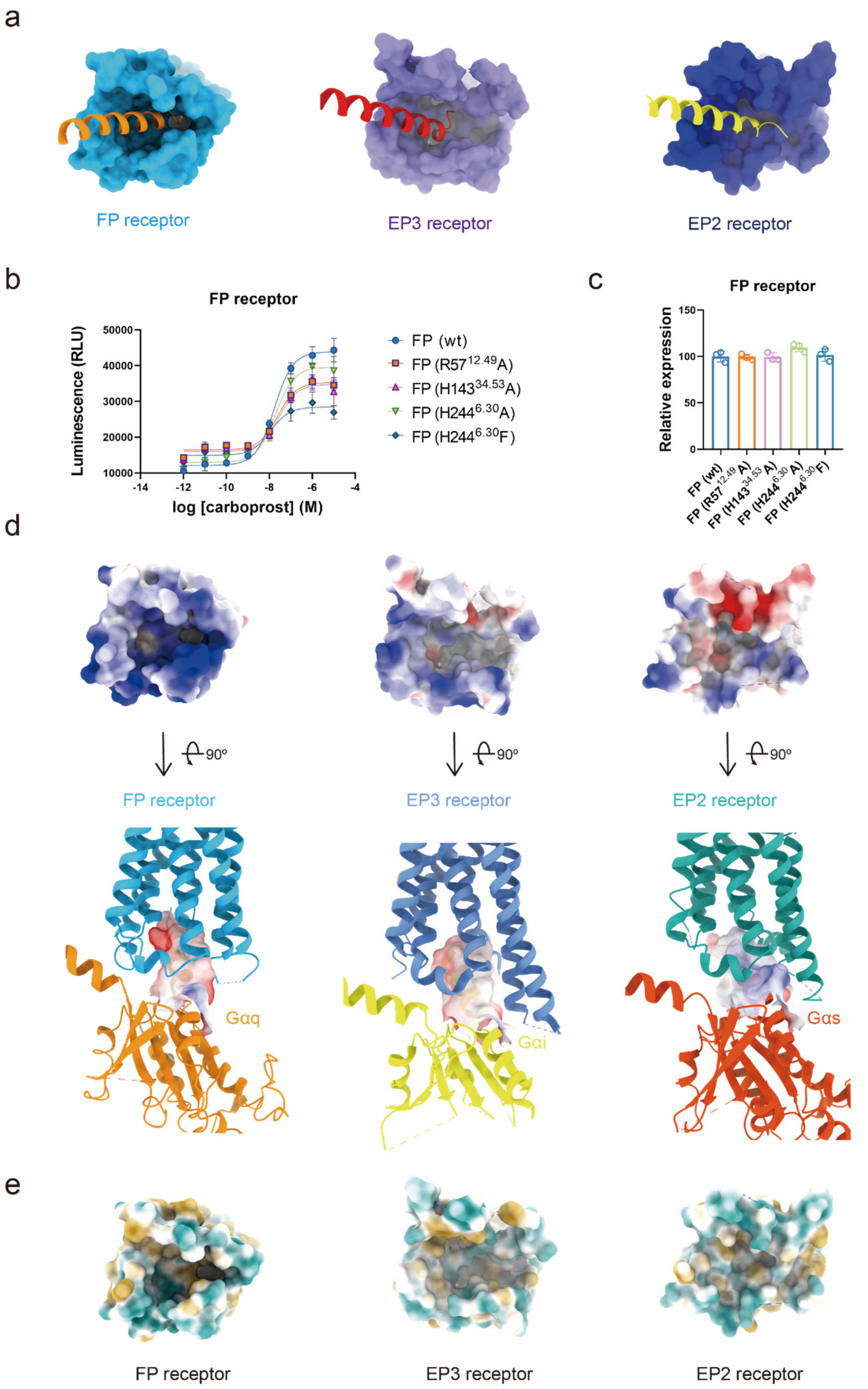

Supplementary Fig. 11 G-protein binding pocket and the interaction pattern

between prostaglandin receptors and G proteins. a Intracellular view of the G protein binding pose bound to the FP receptor, EP3 receptor and EP2 receptor. **b** The carboprost activation profiles of FP receptor and mutants revealed by GloSensor cAMP assay. **c** The expression levels of the R57$^{12.49}$A, H143$^{34.53}$A, H244$^{6.30}$A, H244$^{6.30}$F mutants and wide type FP receptor measured by cell surface staining. Data are given as mean ± SD from 3 independent experiments. **d** The charge distribution of the intracellular G protein binding cavities of the FP receptor, EP3 receptor and EP2 receptor as well as the α5 helices of Gαq, Gαi and Gαs. Blue color indicates positive charge, red color indicates negative charge. **e** The hydrophobicity analysis of the intracellular G protein binding cavities of the FP receptor, EP3 receptor and EP2 receptor. Dark blue indicates hydrophilic surface and darkgolden indicates hydrophobic surface. Source data are provided as a Source Data file.

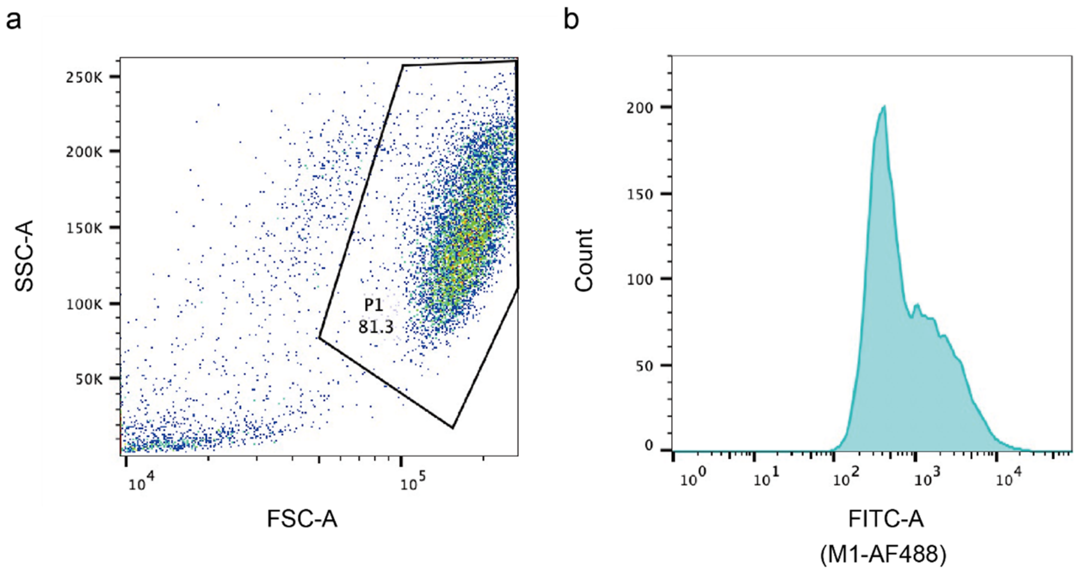

Supplementary Fig. 12 The gating strategy for flow cytometry. a Standard gating strategy based on the size and granularity of the cells is used in the study to select intact cells. b FITC fluorescence distribution of selected cells (P1 in panel a).

Supplementary Table 1

The efficacy of $PGF_{2α}$ and PGE_2 on FP (WT), FP ($S33^{1.39}P$), FP ($H81^{2.54}Q$), FP ($G85^{2.58}T$), EP3 (WT), EP3 ($P55^{1.39}S$), EP3 ($Q103^{2.54}H$) and EP3 ($T107^{2.58}G$).

conditions	ligand	pEC50±SD	ΔpEC50±SD (mutation vs wt)
FP (WT)	$PGF_{2α}$	6.52±0.05	0
FP ($S33^{1.39}P$)		6.65±0.07	0.14±0.08
FP ($H81^{2.54}Q$)		5.73±0.06	-0.80±0.23
FP ($G85^{2.58}T$)		nd	nd
FP (WT)	PGE_2	5.94±0.04	0
FP ($S33^{1.39}P$)		7.04±0.06	1.10±0.04
FP ($H81^{2.54}Q$)		4.57±0.04	-1.39±0.16
FP ($G85^{2.58}T$)		nd	nd
EP3 (WT)	$PGF_{2α}$	5.95±0.09	0
EP3 ($P55^{1.39}S$)		5.38±0.02	-0.57±0.19
EP3 ($Q103^{2.54}H$)		4.19±0.04	-1.76±0.25
EP3 ($T107^{2.58}G$)		6.58±0.03	0.63±0.12
EP3 (WT)	PGE_2	8.65±0.03	0
EP3 ($P55^{1.39}S$)		8.26±0.01	-0.38±0.08
EP3 ($Q103^{2.54}H$)		7.42±0.01	-1.23±0.06
EP3 ($T107^{2.58}G$)		8.48±0.01	-0.16±0.06

"nd" not detectable

Supplementary Table 1| The efficacy of $PGF_{2α}$ and PGE_2 on FP (WT), FP ($S33^{1.39}P$), FP ($H81^{2.54}Q$), FP ($G85^{2.58}T$), EP3 (WT), EP3 ($P55^{1.39}S$), EP3 ($Q103^{2.54}H$) and EP3 ($T107^{2.58}G$). The pEC50 of $PGF_{2α}$ and PGE_2 on FP receptor, EP3 receptor and their mutations as well as the ΔpEC50 of $PGF_{2α}$ and PGE_2 between the mutants and the respective wild-type receptors are shown in the table. Data are given as mean ± SD from 3 independent experiments. Source data are provided as a Source Data file.

Supplementary Table 2

The efficacy of carboprost and latanoprost-FA bound to FP (WT), FP (F265L), EP3(WT) and EP3 (L298F).

conditions	ligand	pEC50±SD	ΔpEC50±SD (mutation vs wt)	# ΔpEC50	comparsion	P-value
FP (wt)	carboprost	7.62± 0.02	-0.80±0.03	#1	1 vs 2	<0.001
FP (F265$^{6.51}$L)		6.82±0.05				
FP (wt)	latanoprost-FA	7.67±0.04	-1.45±0.06	#2		
FP (F265$^{6.51}$L)		6.22±0.04				
EP3 (wt)	carboprost	7.53±0.02	0.36±0.03	#3	3 vs 4	<0.0001
EP3 (L298$^{6.51}$F)		7.89±0.05				
EP3 (wt)	latanoprost-FA	5.04±0.03	1.26±0.04	#4		
EP3 (L298$^{6.51}$F)		6.30±0.01				

Supplementary Table 2| The efficacy of carboprost and latanoprost-FA bound to FP (WT), FP (F265L), EP3(WT) and EP3 (L298F). The pEC50 carboprost and latanoprost-FA bound to FP (WT), FP (F265$^{6.51}$L), EP3 (WT) and EP3 (L298$^{6.51}$F) as well as the ΔpEC50 of carboprost and latanoprost-FA bound to FP (F265$^{6.51}$L) and EP3 (L298$^{6.51}$F) from their equivalent wide-type receptor are shown in the table. The comparison of ΔpEC50 between carboprost and latanoprost-FA bound to FP (F265$^{6.51}$L) from FP (WT) as well as carboprost and latanoprost-FA bound to EP3 (L298$^{6.51}$F) from EP3 (WT) are performed using two tailed Student’s t-test. Data are given as mean ± SD from 3 independent experiments. Source data are provided as a Source Data file.

Supplementary Table 3

Cryo-EM data collection, refinement and validation statistics.

	#1 FP (carboprost) (EMDB-35657) (PDB 8IQ4)	#2 FP (latanoprost) (EMDB-35658) (PDB 8IQ6)
**Data collection and processing**		
Magnification	39000	39000
Voltage (kV)	300	300
Electron exposure (e–/Å^2)	50	50
Defocus range (μm)	1.30-1.80	1.10-1.80
Pixel size (Å)	1.09	1.09
Symmetry imposed	C1	C1
Initial particle images (no.)	2,264,411	1,013,516
Final particle images (no.)	297,293	130,284
Map resolution (Å)	2.8	3.2
FSC threshold	0.143	0.143
Map resolution range (Å)	2.2-3.4	2.5-3.5
**Refinement**		
Initial model used (PDB code)	7RMI	7RMI
Model resolution (Å)	2.7	3.2
FSC threshold	0.143	0.143
Model resolution range (Å)	2.7-3.1	3.3-3.5
Map sharpening *B* factor (Å^2)	-109.8	-132.0
Model composition		
Non-hydrogen atoms	7080	7059
Protein residues	954	954
Ligands	1	1
B factors (Å^2)		
Protein	45.26	40.73
Ligand	56.86	56.48
R.m.s. deviations		
Bond lengths (Å)	0.002 (0)	0.003 (0)
Bond angles (°)	0.487 (0)	0.496 (0)
Validation		
MolProbity score	1.56	1.51
Clashscore	5.38	4.89
Poor rotamers (%)	0.44	0.00
Ramachandran plot		
Favored (%)	0.00	0.00
Allowed (%)	3.98	3.76
Disallowed (%)	96.02	96.24

Supplementary Table 3| Cryo-EM data collection, refinement and validation statistics. The data collection and refinement statistics of FP-miniG$_{s/q70iN}$-Gβ$_1$γ$_2$-Nb35/ carboprost and FP-miniG$_{s/q70iN}$-Gβ$_1$γ$_2$-Nb35/latanoprost-FA structures.
