# Supplementary material for: Structures of human prostaglandin F2α receptor reveal the mechanism of ligand and G protein selectivity
Source: Nat Commun. 2023 Dec 8;14:8136. doi: 10.1038/s41467-023-43922-8 (PMC10709307; doi:10.1038/s41467-023-43922-8)
Supplement: Supplementary file 3 — Reporting Summary [file 41467_2023_43922_MOESM3_ESM.pdf]

nature portfolio

Corresponding author(s): Weishe Zhang; Xiangyu Liu

Last updated by author(s): Nov 22, 2023

Reporting Summary

Nature Portfolio wishes to improve the reproducibility of the work that we publish. This form provides structure for consistency and transparency in reporting. For further information on Nature Portfolio policies, see our Editorial Policies and the Editorial Policy Checklist.

Statistics

For all statistical analyses, confirm that the following items are present in the figure legend, table legend, main text, or Methods section.

n/a	Confirmed	
☐	☒	The exact sample size (*n*) for each experimental group/condition, given as a discrete number and unit of measurement
☐	☒	A statement on whether measurements were taken from distinct samples or whether the same sample was measured repeatedly
☐	☒	The statistical test(s) used AND whether they are one- or two-sided<br>*Only common tests should be described solely by name; describe more complex techniques in the Methods section.*
☒	☐	A description of all covariates tested
☒	☐	A description of any assumptions or corrections, such as tests of normality and adjustment for multiple comparisons
☐	☒	A full description of the statistical parameters including central tendency (e.g. means) or other basic estimates (e.g. regression coefficient) AND variation (e.g. standard deviation) or associated estimates of uncertainty (e.g. confidence intervals)
☐	☒	For null hypothesis testing, the test statistic (e.g. F, t, r) with confidence intervals, effect sizes, degrees of freedom and P value noted<br>*Give P values as exact values whenever suitable.*
☒	☐	For Bayesian analysis, information on the choice of priors and Markov chain Monte Carlo settings
☒	☐	For hierarchical and complex designs, identification of the appropriate level for tests and full reporting of outcomes
☒	☐	Estimates of effect sizes (e.g. Cohen's *d*, Pearson's *r*), indicating how they were calculated

Our web collection on statistics for biologists contains articles on many of the points above.

Software and code

Policy information about availability of computer code

Data collection	FEI Titan Krios 300KV (D3172)
Data analysis	CryoSPARC v3.2; phenix-1.20.1-4487; coot-0.9.8.7; GraphPad Prism 9.1.1; chimeraX-1.3; FlowJo 10.4.0.

For manuscripts utilizing custom algorithms or software that are central to the research but not yet described in published literature, software must be made available to editors and reviewers. We strongly encourage code deposition in a community repository (e.g. GitHub). See the Nature Portfolio guidelines for submitting code & software for further information.

Data

Policy information about availability of data

All manuscripts must include a data availability statement. This statement should provide the following information, where applicable:

- Accession codes, unique identifiers, or web links for publicly available datasets
- A description of any restrictions on data availability
- For clinical datasets or third party data, please ensure that the statement adheres to our policy

Atomic coordinates and structure factors have been deposited in the Protein Data Bank (PDB) under accession codes 8IQ4 and 8IQ6. All the other relevant structures referenced in this work are available under the accession codes 6AK3, 5YWY, 7CX2, 7WU9, 7RMI, 7D7M.

Research involving human participants, their data, or biological material

Policy information about studies with human participants or human data. See also policy information about sex, gender (identity/presentation), and sexual orientation and race, ethnicity and racism.

Reporting on sex and gender	No human research was performed in this study.
Reporting on race, ethnicity, or other socially relevant groupings	Human research participants were not involved in this study.
Population characteristics	Human research participants were not involved in this study.
Recruitment	No human subjects in this study.
Ethics oversight	No human subjects in this study.

Note that full information on the approval of the study protocol must also be provided in the manuscript.

Field-specific reporting

Please select the one below that is the best fit for your research. If you are not sure, read the appropriate sections before making your selection.

☒ Life sciences ☐ Behavioural & social sciences ☐ Ecological, evolutionary & environmental sciences

For a reference copy of the document with all sections, see nature.com/documents/nr-reporting-summary-flat.pdf

Life sciences study design

All studies must disclose on these points even when the disclosure is negative.

Sample size	No statistical methods were used to predetermine sample size. For cryo-EM data, the sample size was chosen based on the sufficient number of particles to achieve high-resolution reconstructions. For the assay, a sample size of n=3 is commonly used in biological studies. All experiments were performed in at least three independent biologial replicates.
Data exclusions	No data were excluded.
Replication	All experiments were repeated at least three times to ensure reproducibility. And all attempts at replication exhibited similar results.
Randomization	Cells placed in different positions on 96-well signaling assay plate and randomly allocated into control and treatment groups.
Blinding	Blinding is not relevant to this study, all experiments were performed based on standardized protocols and readouts and are not influenced by the investigator.

Reporting for specific materials, systems and methods

We require information from authors about some types of materials, experimental systems and methods used in many studies. Here, indicate whether each material, system or method listed is relevant to your study. If you are not sure if a list item applies to your research, read the appropriate section before selecting a response.

Materials & experimental systems

n/a	Involved in the study	
☐	☒	Antibodies
☐	☒	Eukaryotic cell lines
☒	☐	Palaeontology and archaeology
☒	☐	Animals and other organisms
☒	☐	Clinical data
☒	☐	Dual use research of concern
☒	☐	Plants

Methods

n/a	Involved in the study	
☒	☐	ChIP-seq
☐	☒	Flow cytometry
☒	☐	MRI-based neuroimaging

Antibodies

Antibodies used	Alexa-488 conjugated anti-Flag antibody (Thermo Fisher, Cat # MA1-142-A488)

Validation	The antibody was commercially available and validated by the manufacturer. The website is https://www.thermofisher.cn/cn/zh/antibody/product/DYKDDDDK-Tag-Antibody-clone-L5-Monoclonal/MA1-142-A488.

Eukaryotic cell lines

Policy information about cell lines and Sex and Gender in Research

Cell line source(s)	Sf9 cells were purchased from Expression Systems, Cat 94-0015. Cos7 cells (ATCC CRL-1651) were purchased from ATCC.
Authentication	Cos7 cells have been validated by the supplier through STR analysis and cytogenetic studies. Except for cos7 cells, no authentication required.
Mycoplasma contamination	Cell lines are tested by manufacturer for contamination and no additional test was conducted by authors of this study.
Commonly misidentified lines (See ICLAC register)	No commonly misidentified cell lines were used.

Plants

Seed stocks	No plant research was performed in this study.
Novel plant genotypes	No plant research was performed in this study.
Authentication	The study did not involve plants.

Flow Cytometry

Plots

Confirm that:

☒ The axis labels state the marker and fluorochrome used (e.g. CD4-FITC).

☒ The axis scales are clearly visible. Include numbers along axes only for bottom left plot of group (a 'group' is an analysis of identical markers).

☒ All plots are contour plots with outliers or pseudocolor plots.

☒ A numerical value for number of cells or percentage (with statistics) is provided.

Methodology

Sample preparation	The transfected cells used for the functional assay were used for staining. In brief, the cells were resuspended in HBSS supplemented with 20 mM HEPES, pH 7.5 and incubated with Alexa-488 conjugated anti-Flag antibody in the dark for 15 minutes at room temperature. Cells were washed twice before the expression levels were detected by flow cytometry.
Instrument	BD LSRFortessa
Software	FlowJo 10.4.0.
Cell population abundance	Not applicable to the flow analysis.
Gating strategy	Standard gating strategy was used based on the size and granularity of the cells and gating strategy was shown in the supplementary information.

☒ Tick this box to confirm that a figure exemplifying the gating strategy is provided in the Supplementary Information.
